# A reproducible R workflow for harmonizing indoor air quality sensor data and cleaning activity logs in school-based field campaigns^☆^

**DOI:** 10.1016/j.mex.2026.103995

**Published:** 2026-06-05

**Authors:** C. Falzone, H. Moujahid, N. Redon, F. Thevenet, M. Verriele

**Affiliations:** a IMT Nord Europe, Université de Lille, CERI EE, Lille F-59000, France; b Research Unit SPHERE, Department of Environmental Sciences and Management, University of Liege, Liège 4000, Belgium

**Keywords:** Occupational exposure, Wearable sensors, Time–activity data, Data harmonization, Sensor preprocessing, Data visualization

## Abstract

This article presents a reproducible workflow developed in R to process indoor air quality data by integrating wearable multi sensor measurements with tablet-based activity logs. The data come from two field campaigns in four primary schools and involve 20 female maintenance staff members. The method supports occupational exposure assessment under real world conditions, where sensor signals can be incomplete or noisy and self-reported activities may contain timing errors. The workflow organizes raw files by campaign, school and instrument, standardized timestamps (including daylight saving changes), and harmonizes quantitative signals (temperature, relative humidity, CO_2_, PM_2.5_ and VOC sensors) with qualitative descriptors from activity logs (location, activity, cleaning practice, product, time). It then builds an integrated dataset in which each sensor reading is linked to its activity context, making it possible to distinguish cleaning periods from daytime and night time storage of instruments. The approach produces graphical outputs and summary statistics that support sensor diagnostics, comparison of exposure profiles between micro environments, and communication of semi quantitative exposure patterns to researchers and stakeholders.

• Reproducible R workflow integrating multisensor and activity data.

• Standardized pipeline for data cleaning, temporal alignment, and data structuring.

• Analysis-ready outputs linking exposure levels to activities and contexts.

## Graphical abstract

**Figure:**
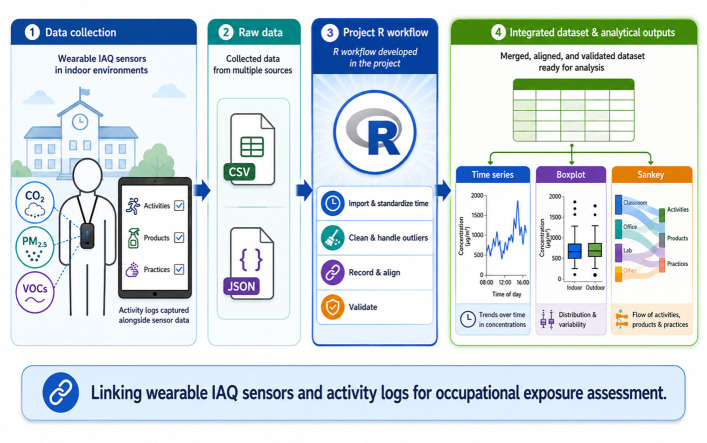

## Specifications table

**Table:** 

**Subject area**	Environmental Science
**More specific subject area**	Indoor air quality; occupational exposure assessment
**Name of your method**	A R-based workflow for integrating multi-sensor indoor air quality data with cleaning activity logs
**Name and reference of original method**	Not applicable
**Resource availability**	OEEIL monitoring sensor device: https://anemon-sensors.com/en/nos-produits/ The workflow is implemented in R using open-source packages (tidyverse, lubridate, ggplot2, jsonlte, netwokD3, webshot, openair).

## Background

Recent developments in wearable air quality monitoring highlight their growing role not only as measurement tools but also as potential levers for awareness and empowerment, by making otherwise invisible exposures tangible at the individual scale [1]. In indoor environments, sensor feedback has been explored as a means to improve understanding of pollutant sources and to support behavioral adaptation [2]. Access to sensor-based information can enhance awareness of exposure situations and everyday determinants such as ventilation, cleaning, or occupancy patterns, although the translation into behavioral change may remain strongly context-dependent. The relationship between environmental exposure and health is mediated by perceptions of the exposure. While sensors generate large volumes of individualized data, their capacity to support empowerment may depend on how these data are structured, interpreted, and made intelligible to users [3]. Studies on indoor air quality combining measurements from wearable sensor with activity diaries may generate rich but structurally heterogeneous datasets. Semi-quantitative observations are typically recorded at short time intervals and are strongly time dependent [4], whereas qualitative logs are event-based, may contain multiple entries for a single activity, and often differ in temporal reference and level of detail.

In this context, data processing is a key step that directly determines the validity, interpretability, and usability of the results. Harmonizing these datasets cannot be done with simple spreadsheet operations; it requires scripted tools such as R and a good understanding of sensor behavior, data quality and the field context. Beyond their structural heterogeneity, these datasets are affected by multiple sources of variability and degradation inherent to real-world field conditions. These include human factors (e.g., incomplete or imprecise activity logs, inconsistent time reporting, improper handling of devices), professional context constraints (e.g., absences, variable adherence to protocols, heterogeneous storage conditions outside activity periods), technical limitations (e.g., sensor drift, signal noise, missing data, device malfunction, inconsistencies between instruments), and external or unforeseen events (e.g., daylight saving time changes, network interruptions, data transmission failures). Taken together, these factors can substantially alter the continuity, coherence, and interpretability of time series, and thus require explicit and methodologically sound processing strategies.

The present work was therefore designed to provide a clear, reproducible, and fully documented R workflow for harmonizing, cleaning, and merging these heterogeneous data sources in real-world field conditions. The study was conducted within the EXAMEN project, funded by ANSES through the National Research Program for Environmental and Occupational Health (PNR EST, grant no. EST-2023-145), in which two measurement campaigns were carried out in four primary schools in Nancy (Grand Est region – France). During each campaign, cleaning staff were equipped with portable indoor air quality monitoring instruments, referred to as OEEIL, previously described [5], worn at torso level, while they simultaneously completed tablet-based activity logs.

## Method details

The OEEIL device is a lightweight (∼400 g), battery-powered system designed for individual exposure assessment (e.g. minimizing real-time user interaction in order to limit behavioral bias). It integrates sensors for temperature, relative humidity, CO₂ (NDIR), particulate matter (optical counter), and gas-phase pollutants including NO₂, O₃, and volatile organic compounds (MOX-based sensors). Data are continuously recorded and transmitted to a secure platform.

The activity logs recorded the location, activity performed, cleaning practice, product used, start time, and duration of each task. The campaigns captured measurements over several days and distinguished between cleaning activity periods, inactive periods, and storage periods. As expected in real-world field conditions, the datasets were also affected by common field constraints, including participant absences, partially completed or incorrectly filled logs, interrupted measurements, and instrument malfunctions. The workflow was designed to incorporate these real-world limitations without forcing the data into an artificial or inappropriate structure.

The quantitative dataset included time-stamped measurements of temperature, relative humidity, CO_2_, PM_2.5_, and gas sensor outputs associated with NO_2_, O_3_, and volatile organic compounds. The qualitative dataset was exported in JSON format and reorganized into standardized tables with anonymized identifiers. The R script automates the import of both datasets, the conversion of timestamps to a unified time scale, the correction of daylight saving time, the handling of missing values and sensor error codes, and the creation of composite activity categories when multiple products or practices were associated with a single event. This approach improves traceability and facilitates the comparison of measurements collected during activity, daytime storage, and nighttime storage periods. Several R packages were used in the development of the code, particularly for data import, manipulation, visualization, and date-time handling. A summary of the main packages retained in the final version of the script is provided in Table 1.

**Table 1:** R packages used in the final version of the workflow.

Package	Main use in the workflow	Citation
dplyr	Data wrangling and summarising	Wickham et al. [6]
tidyr	Data reshaping	Wickham et al. [7]
jsonlite	Import of activity logs	Ooms [8]
lubridate	Date-time handling	Grolemund et Wickham [9]
ggplot2	Time-series and boxplot plotting	Wickham [10]
networkD3	Sankey diagrams	Allaire et al. [11]
webshot	Export of interactive figures HTLM to PNG	Chang [12]
openair	Air quality analysis and visualization	Carslaw et Ropkins [13]

The main contribution of this article lies in the development of a code written in R, enabling reproducible data processing that can be adapted for use in other exposure studies. Its purpose is to link indoor environmental measurements with contextual activity data. It is particularly relevant for field studies involving repeated campaigns, conducted at one or more sites, incorporating both data from heterogeneous sensors and behavioral information from activity log. By documenting each processing step in detail, the article supports transparency, reuse, and comparability across indoor air quality studies.

### Study design and datasets

Two measurement campaigns were conducted in four primary schools. Each campaign involved several cleaning staff members, anonymized by an instrument identifier, with no names or directly identifiable information reported. The monitoring instruments were worn during working hours only, while the staff simultaneously recorded their activities on individual tablets.

The quantitative dataset consisted of time-stamped time series produced by the monitoring instruments. The main variables retained for analysis were temperature, relative humidity, CO_2_, PM_2.5_, and VOC. The qualitative dataset contained the information entered by the staff during their tasks: cleaning location, activity performed, practice used, product applied, start time, and duration. The qualitative data were recorded in JSON format, whereas the quantitative data were exported in CSV format.

The two campaigns were not strictly identical. Differences concerned exposure duration, the number of days the instruments were worn, the set of usable days, and the availability of qualitative data. The workflow was therefore designed to remain identical in its overall logic while allowing minor adjustments according to the specific characteristics of each campaign.

### Data import and organization

**Fig. 1:**
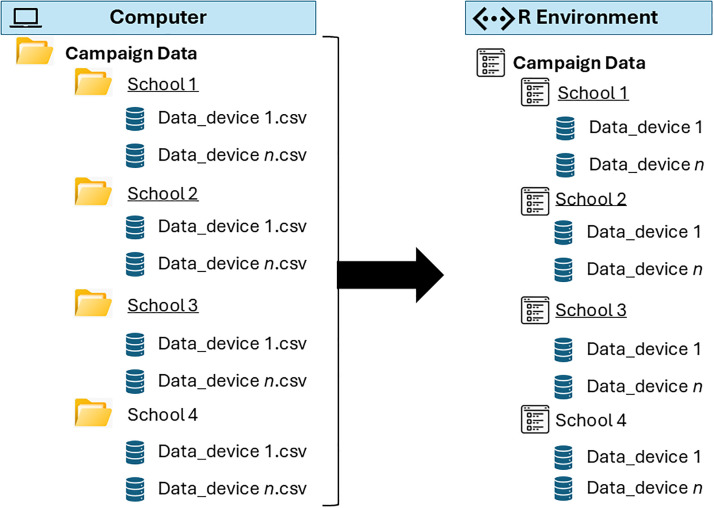
File organization for automation (import and processing). JSON files follow the same structure.

The raw files were organized hierarchically by campaign, school, and instrument or participant. This organization was preserved during import to maintain full data traceability. In the R script, the CSV and JSON files were automatically read from predefined directories and stored in list-based structures, with one list per school and, within each list, one object per instrument or participant. (Fig. 1)

This import step had a dual purpose. First, it standardized the reading of multiple files without requiring manual opening one by one. Second, it facilitated anonymization, since the objects created in the R environment were named according to technical logic rather than the participants’ identities. The choice of a list structure also made it possible to preserve the data hierarchy while simplifying the automation of subsequent processing steps.

### Time handling and harmonization

Temporal harmonization was a central step of the workflow because the quantitative and qualitative datasets were not recorded in the same format and did not always follow the same time reference. The sensor data were converted into a standardized date-time format using POSIX-compatible objects. The activity logs, which were recorded in local time, were also converted into a unified time scale to enable alignment with the sensor measurements.

Special attention was given to daylight saving time changes occurring during the campaign period. When necessary, time stamps affected by the clock change were adjusted manually by adding the corresponding number of seconds to preserve chronological continuity. This step was essential to avoid mismatches between activity records and environmental measurements. After time conversion, the data were reorganized so that each observation could be correctly linked to its corresponding temporal interval.

### Quantitative data processing

The quantitative dataset included several variables measured at regular intervals. For this work, only the variables useful for exposure interpretation were retained. The limited metrological performance of the OEEIL devices, characterized by cross-sensitivity, signal drift, and environmental interferences, is acknowledged and explicitly accounted for in the data processing strategy. The signals generated cannot be interpreted as accurate pollutant concentrations but rather as semi-quantitative indicators of exposure dynamics [14].

The columns related to date, temperature, relative humidity, CO_2_, PM_2.5_, and the conductance from VOC sensors were kept in the final structure.

The data were then cleaned according to explicit rules. Some values were identified as instrument error codes and replaced with missing values (Table 2). For example, extreme values corresponding to sensor error messages were excluded from the analyzed dataset. Similarly, some unusually high values observed for metal-oxide sensors were considered aberrant and converted to missing values. This treatment helped reduce analytical noise and ensured that the subsequent graphs and analyses were based on interpretable values.

**Table 2:** Outliers or threshold values for the various sensors.

Sensors	Output sensor
CO_2_	65,535 ppm
Relative Humidity	655 %RH
VOC - Metal oxide	>10^7^

The purpose of this step was not only to clean the data, but also to identify instruments or periods showing recurrent malfunctions. Time-series plots and summary statistics were used to quickly detect problematic sensors, visualize signal disruptions, and verify the overall consistency of the measurements.

### Qualitative data processing

The activity logs are an essential complement for interpreting pollution variations measured during work. They contain information on the activity performed, the location, the cleaning practice used, and the products employed. Because a single activity could be associated with several products or several practices, a restructuring step was necessary to make these data usable in temporal and relational analyses.

**Fig. 2:**
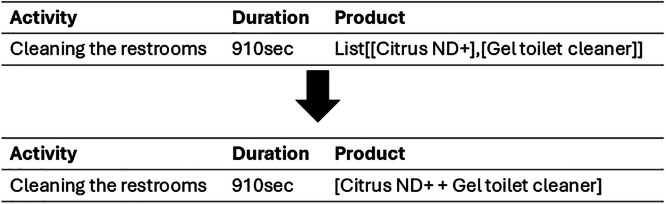
Schematic illustration of recoding multiple entries within a single variable into a single harmonized code, to avoid list-type fields in the R environment.

The variables were first harmonized so that common labels could be used across campaigns (Fig. 2). Time fields were then converted into a homogeneous format and mapped onto a common timestamp compatible with the sensor measurements. Composite categories were subsequently created for activities involving multiple practice-product combinations. This choice made it possible to preserve the information collected in the field while simplifying statistical and graphical analysis.

### Data alignment and merging

Once the two datasets were standardized, the next step was to merge them on the basis of time. The principle adopted was to match the reported activity windows with the sensor measurements recorded during the same period. To do this, activities were treated as events extending over a defined duration, which made it possible to link each qualitative record to the environment measured at that same period of time.

**Fig. 3:**
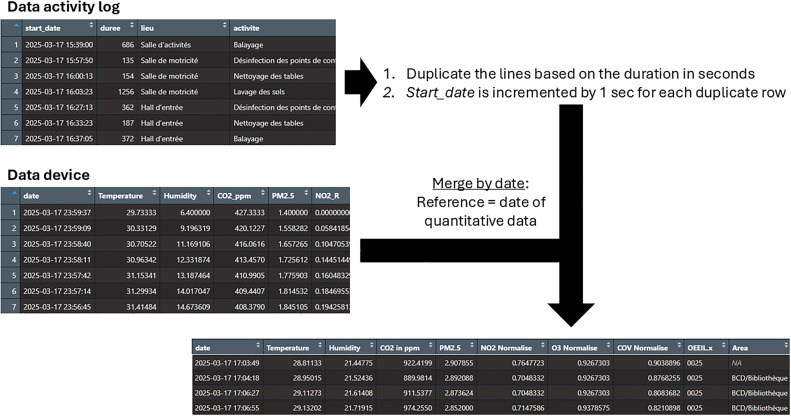
Strategy for combining the two datasets: data from activity log and device multisensors.

This merge required several intermediate operations. The qualitative data were adapted so that, for each activity, a time sequence compatible with the temporal granularity of the quantitative data could be generated (Figure 5). The merged datasets then made it possible to distinguish between active work periods, daytime storage periods, and nighttime storage periods. This distinction is important because it allows comparison of the signals observed during cleaning activities with those observed outside these activities. (Fig. 3)

The temporal merge transformed two heterogeneous sources into an integrated dataset in which each sensor observation could be interpreted in light of the corresponding activity context. This step then made it possible to compare exposure patterns and to analyze the associations between tasks, practices, and measured emissions.

### Handling missing data and field constraints

The campaigns were conducted under real field conditions, which inevitably led to missing information. Some participants were absent on certain days, some logs were incomplete, and some measurements were affected by technical incidents. The workflow was therefore designed to filter the data according to the days that were usable rather than treating all observations as equivalent.

Selection rules were applied to retain only sequences that were coherent between sensor measurements and activity logs. This approach helps limit bias related to absences, measurement interruptions, and instrument-related problems. It also facilitates comparison between campaigns, as it is based on explicit and reproducible selection criteria.

### Output generation and visualization

Data visualization plays a central role in this work. It does not only used to illustrate results, but also helps make the dataset interpretable and supports a coherent narrative from complex and heterogeneous data. Without this step, the data remains fragmented and difficult to relate to real-world situations or exposure scenarios. Several types of representations were therefore prioritized according to their ability to address specific methodological questions.

**Fig. 4:**
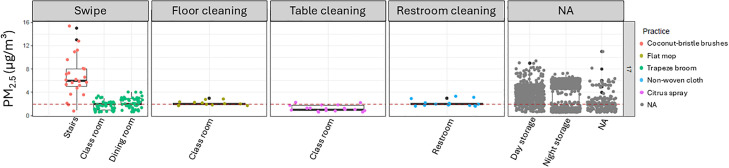
Distribution of mass concentration of PM_2.5_ by day, activities, area and practice for one person in one school.

Time-series plots were used first to inspect the quality of the raw and cleaned signals. They make it possible to quickly identify anomalies, gaps in measurement, drifts, and stable periods. Boxplots were retained to compare signal distributions across periods, categories, or instruments (Fig. 4). They are particularly useful for visualizing differences in central tendency and dispersion without overloading the reader.

**Fig. 5:**
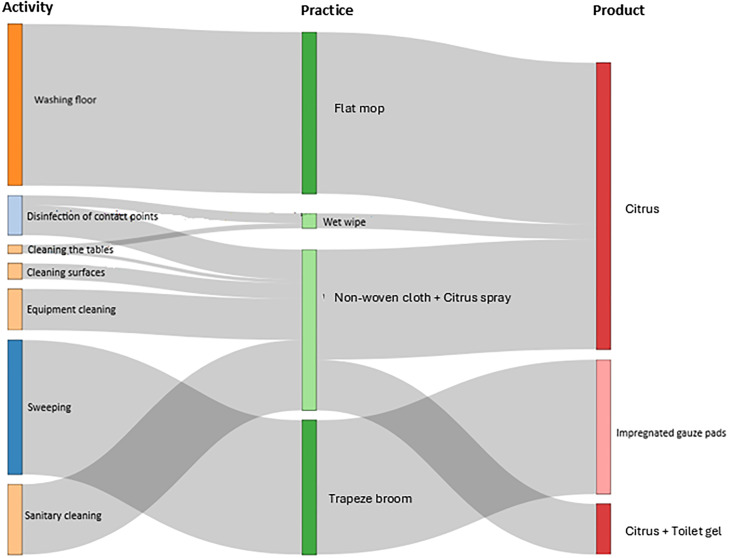
A Sankey diagram linking activities to practices and products used. The width of each bar is proportional to the time spent. Groups together all activities performed by an agent during a campaign.

Sankey diagrams are probably the most informative representation for qualitative data. In this work, they were used to link activities to cleaning practices and then to the products used (Fig. 5). Because the thickness of the flows is proportional to cumulative duration, this type of graphic makes it possible to rapidly identify the most frequent combinations and, therefore, the sequences that are potentially most relevant in terms of exposure. The Sankey diagram thus became an essential tool for visually ranking the relationships between activity, practice, and product.

Specific graphs were also generated to distinguish non-activity periods, daytime and nighttime storage periods, and comparisons between active and rest periods. Finally, summary statistics were produced to document both the raw and cleaned datasets by reporting minimum and maximum values, quartiles, means, and numbers of missing values.

### Reproducibility and code structure

The entire workflow was implemented in R and divided into successive sections to improve readability and modularity. Each code block corresponds to a specific processing step, such as data loading, time handling, outlier management, activity categorization, or output generation. This structure made the workflow easier to adapt between campaigns while keeping the logic identical.

The script was written so that the same processing principles could be applied to both campaigns, with only minor adjustments required for campaign-specific features. All path definitions were externalized to facilitate reuse in other environments, and the processing logic was kept transparent through explicit variable names and commented sections. This design supports reproducibility and makes the workflow suitable for reuse in similar indoor air quality studies.

### Reuse potential

This workflow can be reused in any context where indoor air quality measurements are collected simultaneously with activity logs. It is particularly relevant for exposure studies based on wearable sensors, field observations, and behavioral data. Its modular structure also makes it adaptable to other campaigns, provided that the data are organized around a common time axis. By supporting the translation of complex, high-resolution measurements into accessible representations, it enables the organization of collective exchange sessions aimed at co-constructing recommendations and fostering reflection on behavioral change.

Beyond the case study presented here, the main value of this approach is that it offers a transparent method for transforming heterogeneous raw data into an analytically usable dataset. In practice, the workflow behaves as a methodological tool rather than as a single analysis script. It can be adapted to other studies on indoor air quality, ventilation, occupational exposure or cleaning practices that rely on time-resolved sensor data and activity information.

## Method validation

The workflow was validated on two multi-day campaigns in four primary schools. Time-series plots and summary statistics systematically revealed sensor malfunctions and inconsistent signals, allowing faulty devices and extreme outliers to be excluded from the analysis. After cleaning and temporal alignment, the merged datasets showed coherent patterns across campaigns, with exposure dynamics that were consistent with reported activities, storage periods, and known device limitations. The visualization suite (time-series, boxplots, and Sankey diagrams) further confirmed the internal consistency of the processed data by revealing plausible relationships between tasks, practices, products, and semi-quantitative exposure indicators.

### Limitations

The proposed workflow is tailored to semi-quantitative, low-cost multi-sensor devices and should not be used to derive accurate pollutant concentrations or to assess compliance with health-based guideline values. Its performance depends on the availability and quality of activity logs; incomplete or inaccurate data therefore reduce the reliability of the temporal alignment between activities and sensor signals. Several processing choices (e.g., outlier thresholds, activity recoding rules, storage period definitions) are project-specific and should be re-evaluated before applying the workflow to other populations or monitoring devices. Finally, the method assumes that all data sources share a common timestamp. Consequently, applications that do not fit this type of temporal design would require significant code adaptation.

## Ethics statements

All procedures involving human participants complied with established ethical standards for research on personal exposure monitoring. Volunteers were recruited on a strictly voluntary basis, without any hierarchical influence or obligation to participate. Prior to inclusion, all participants attended an information session detailing the study objectives, measurement protocol, and data protection measures. Each participant received a written information sheet and provided signed informed consent before any data collection. Participants were informed of their right to withdraw at any time without justification. Strict data protection measures were implemented in compliance with the General Data Protection Regulation and the French Data Protection Act. Personal data were pseudonymised, securely stored, and accessible only to authorised members of the research team. Sensor data, including potential geolocation and activity-related information, were treated as confidential and were not shared with participants’ employers or hierarchy. Only aggregated and anonymised results were disseminated. Participants were explicitly informed of data processing procedures, storage duration, and their rights (access, rectification, erasure).

## Declaration of generative AI and AI-assisted technologies in the manuscript preparation process

During the preparation of this work the author(s) used Perplexity and ChatGPT in order to improve the clarity and quality of the English language. After using this tool/service, the author(s) reviewed and edited the content as needed and take(s) full responsibility for the content of the published article.

## CRediT authorship contribution statement

**C. Falzone:** Methodology, Software, Validation, Data curation, Visualization, Writing – original draft. **H. Moujahid:** Investigation. **N. Redon:** Resources, Investigation. **F. Thevenet:** Conceptualization, Writing – review & editing, Supervision, Funding acquisition. **M. Verriele:** Conceptualization, Writing – review & editing, Supervision, Project administration, Funding acquisition.

## Declaration of competing interest

The authors declare that they have no known competing financial interests or personal relationships that could have appeared to influence the work reported in this paper.

## Data Availability

Data will be made available on request.
